# Macrophage Specific Caspase-1/11 Deficiency Protects against Cholesterol Crystallization and Hepatic Inflammation in Hyperlipidemic Mice

**DOI:** 10.1371/journal.pone.0078792

**Published:** 2013-12-02

**Authors:** Tim Hendrikx, Veerle Bieghs, Sofie M. A. Walenbergh, Patrick J. van Gorp, Fons Verheyen, Mike L. J. Jeurissen, Mandy M. F. Steinbusch, Nathalie Vaes, Christoph J. Binder, Ger H. Koek, Rinke Stienstra, Mihai G. Netea, Marten H. Hofker, Ronit Shiri-Sverdlov

**Affiliations:** 1 Departments of Molecular Genetics, Electron Microscopy and Internal Medicine, Nutrition and Toxicology Research (NUTRIM) Institute of Maastricht and Maastricht University Medical Centre (MUMC), Maastricht University, Maastricht, The Netherlands; 2 Department of Laboratory Medicine, Medical University of Vienna, Vienna, Austria; 3 Center for Molecular Medicine, Austrian Academy of Sciences, Vienna, Austria; 4 Department of Medicine, Radboud University Nijmegen Medical Centre and Nijmegen Institute for Infection, Inflammation and Immunity (N4i), Nijmegen, The Netherlands; 5 Department of Human Nutrition, Wageningen University, Wageningen, The Netherlands; 6 Department of Pathology and Medical Biology, Molecular Genetics, University of Groningen, University Medical Center Groningen, Groningen, The Netherlands; Virginia Tech University, United States of America

## Abstract

**Background & Aims:**

While non-alcoholic steatohepatitis (NASH) is characterized by hepatic steatosis combined with inflammation, the mechanisms triggering hepatic inflammation are unknown. In Ldlr^-/-^ mice, we have previously shown that lysosomal cholesterol accumulation in Kupffer cells (KCs) correlates with hepatic inflammation and cholesterol crystallization. Previously, cholesterol crystals have been shown to induce the activation of inflammasomes. Inflammasomes are protein complexes that induce the processing and release of pro-inflammatory cytokines IL-1b and IL-18 via caspase-1 activation. Whereas caspase-1 activation is independent of caspase-11 in the canonical pathway of inflammasome activation, caspase-11 was found to trigger caspase-1-dependent IL-1b and IL-18 in response to non-canonical inflammasome activators. So far, it has not been investigated whether inflammasome activation stimulates the formation of cholesterol crystals. We hypothesized that inflammasome activation in KCs stimulates cholesterol crystallization, thereby leading to hepatic inflammation.

**Methods:**

*Ldlr* ^*-/-*^ mice were transplanted (tp) with wild-type (Wt) or caspase-1/11^-/-^ (dKO) bone marrow and fed either regular chow or a high-fat, high-cholesterol (HFC) diet for 12 weeks. *In* *vitro*, bone marrow derived macrophages (BMDM) from wt or caspase-1/11^-/-^ mice were incubated with oxLDL for 24h and autophagy was assessed.

**Results:**

In line with our hypothesis, caspase-1/11^-/-^-tp mice had less severe hepatic inflammation than Wt-tp animals, as evident from liver histology and gene expression analysis in isolated KCs. Mechanistically, KCs from caspase-1/11^-/-^-tp mice showed less cholesterol crystals, enhanced cholesterol efflux and increased autophagy. In wt BMDM, oxLDL incubation led to disturbed autophagy activity whereas BMDM from caspase-1/11^-/-^ mice had normal autophagy activity.

**Conclusion:**

Altogether, these data suggest a vicious cycle whereby disturbed autophagy and decreased cholesterol efflux leads to newly formed cholesterol crystals and thereby maintain hepatic inflammation during NASH by further activating the inflammasome.

## Introduction

Non-alcoholic fatty liver disease (NAFLD) constitutes a spectrum of liver diseases characterized by hepatic lipid accumulation (steatosis), which when combined with hepatic inflammation is known as non-alcoholic steatohepatitis (NASH). While steatosis itself is generally considered a rather harmless and reversible condition that is present in about two thirds of patients with metabolic syndrome, the presence of inflammation in a fatty liver drives disease progression and allows the disease to develop to more advanced stages – such as fibrosis, cirrhosis or hepatocellular carcinoma – ultimately requiring liver transplantation [1]. Currently, the mechanisms underlying hepatic inflammation are unknown. Consequently, therapeutic options are poor and non-invasive markers to detect NASH do not exist. It is therefore important to investigate the underlying mechanisms that trigger the hepatic inflammatory response in the progression to NASH.

Previously, in mice lacking the low-density lipoprotein receptor (*Ldlr* ^*-/-*^), we have shown that hepatic inflammation is initiated by Kupffer cells (KCs) [2], which scavenge oxidized lipoproteins rich in cholesterol, leading to an accumulation of cholesterol inside the lysosomes of KCs and the formation of cholesterol crystals [3,4]. Cholesterol crystals have been shown to induce the activation of inflammasomes [5]. Inflammasomes are large multi-protein complexes that, via caspase-1 activation, trigger the maturation of the pro-inflammatory cytokines interleukin-1b (IL-1b) and interleukin-18 (IL-18), which then engage in innate immune defense and sustain inflammation [6–8]. Whereas caspase-1 activation is independent of caspase-11 in the canonical pathway of inflammasome activation, pro-inflammatory caspase-11 was found to be essential for LPS-induced IL-1b secretition *in vivo* [9] and trigger caspase-1 dependent IL-1b and IL-18 in response to non-canonical inflammasome activators [10]. So far, the contribution of hematopoietic inflammasome activation to the formation of cholesterol crystals has not been investigated. We hypothesized that inflammasome activation in KCs stimulates the formation of cholesterol crystals, thereby leading to inflammation in the liver.

To test this hypothesis, we transferred bone marrow cells from donor mice lacking caspase-1 and caspase-11 (dKO) into lethally irradiated hyperlipidemic Ldlr^-/-^ recipient mice. In line with our hypothesis, when these mice were fed an HFC diet, deletion of caspase-1/11 in KCs resulted in less hepatic inflammation compared to controls, which was independent of the development of hepatic steatosis. Interestingly, lysosomal dysfunction and cholesterol crystallization inside KCs was lower in caspase-1/11^-/-^-tp mice on HFC diet than in Wt-tp mice. Mechanistically, we show that cholesterol efflux and autophagy are involved in inflammasome induced cholesterol crystallization. Additionally, we strengthen our *in vivo* findings *in vitro*, as BMDM from Wt mice had affected autophagic activity whereas caspase-1/11^-/-^ BMDM had normal autophagic activity upon oxLDL incubation. These data suggest a vicious cycle whereby disturbed autophagy and decreased cholesterol efflux leads to newly formed cholesterol crystals that further enhance the activation of the inflammasome and thereby maintain hepatic inflammation. Taken together, our data propose that inflammasome activation contributes to the sustained inflammatory response in the liver via the formation of cholesterol crystals. Therefore, inhibiting the inflammatory cascade induced by inflammasome activation in KCs or abrogating the cholesterol crystallization process by restoring autophagy or cholesterol efflux may be beneficial for the treatment of NASH.

## Methods

### Mice, diet and bone marrow transplantation

Mice were housed under standard conditions and given unlimited access to food and water. Experiments were performed according to Dutch regulations and approved by the Committee for Animal Welfare of Maastricht University. Female 10-12 week old *Ldlr* ^*-/-*^ mice were lethally irradiated and transplanted with Wt or caspase-1/11^-/-^ bone marrow as previously described [2]. Caspase-1/11^-/-^ mice were a kind gift from Prof. Netea, Department of Medicine, Radboud University Nijmegen Medical Centre and Nijmegen Institute for Infection, Inflammation and Immunity (N4i), Nijmegen, The Netherlands. All mice were backcrossed ten generations to C57Bl/6J mice, and age-matched wild-type C57Bl/6J mice were used as controls throughout the different experiments. Besides caspase-1, these mice are known to have defective caspase-11, essentially making them doubly deficient in caspase-1 and caspase-11 [10]. Efficiency of the bone marrow transplantation was approximately 98% (data not shown). After 9 weeks of recovery, mice were given either chow or HFC diet for 3 months (Wt-tp chow: n=11; HFC: n=12; caspase-1/11^-/-^-tp chow: n=11; HFC: n=14). The HFC diet contained 21% milk butter, 0.2% cholesterol, 46% carbohydrates and 17% casein. Collection of blood and tissue specimens, biochemical determination of plasma and liver lipids, liver histology, electron microscopy, Kupffer cell isolation, cathepsin D activity assay, *in vitro* macrophage culture, western blotting, RNA isolation, cDNA synthesis and qPCR were performed as described previously [2–4,11–13]. 

### Autophagy analysis

In electron microscopy pictures autophagy analysis was performed by counting lipid droplets with a double membrane (autophagosomes) in 40 Kupffer cells from each transplanted group and calculating the average autophagosome number per Kupffer cell. In order to score cholesterol crystallization in Kupffer cells, electron microscopy pictures of 30 Kupffer cells from each experimental group were investigated for the amount of cholesterol crystals present. Each Kupffer cell was given a score in the range 0 to 5; 0 indicates that no cholesterol crystals were present while 5 indicates the highest amount of cholesterol crystals present.

### Statistical analysis

The data were analyzed using Graphpad Prism 4.0.3 (GraphPad Software, Inc., La Jolla, CA, USA). The unpaired t-test was performed for comparing Wt-tp and caspase-1/11^-/-^-tp mice for each diet group and for comparing Wt and caspase-1/11^-/-^ BMDM. The data were expressed as the mean and standard error of the mean (SEM) and were considered significantly different at *p<0.05; ** p<0.01; *** p<0.001.

## Results

### Hematopoietic deletion of caspase-1/11 has no effect on plasma and liver lipid levels

**Figure 1:**
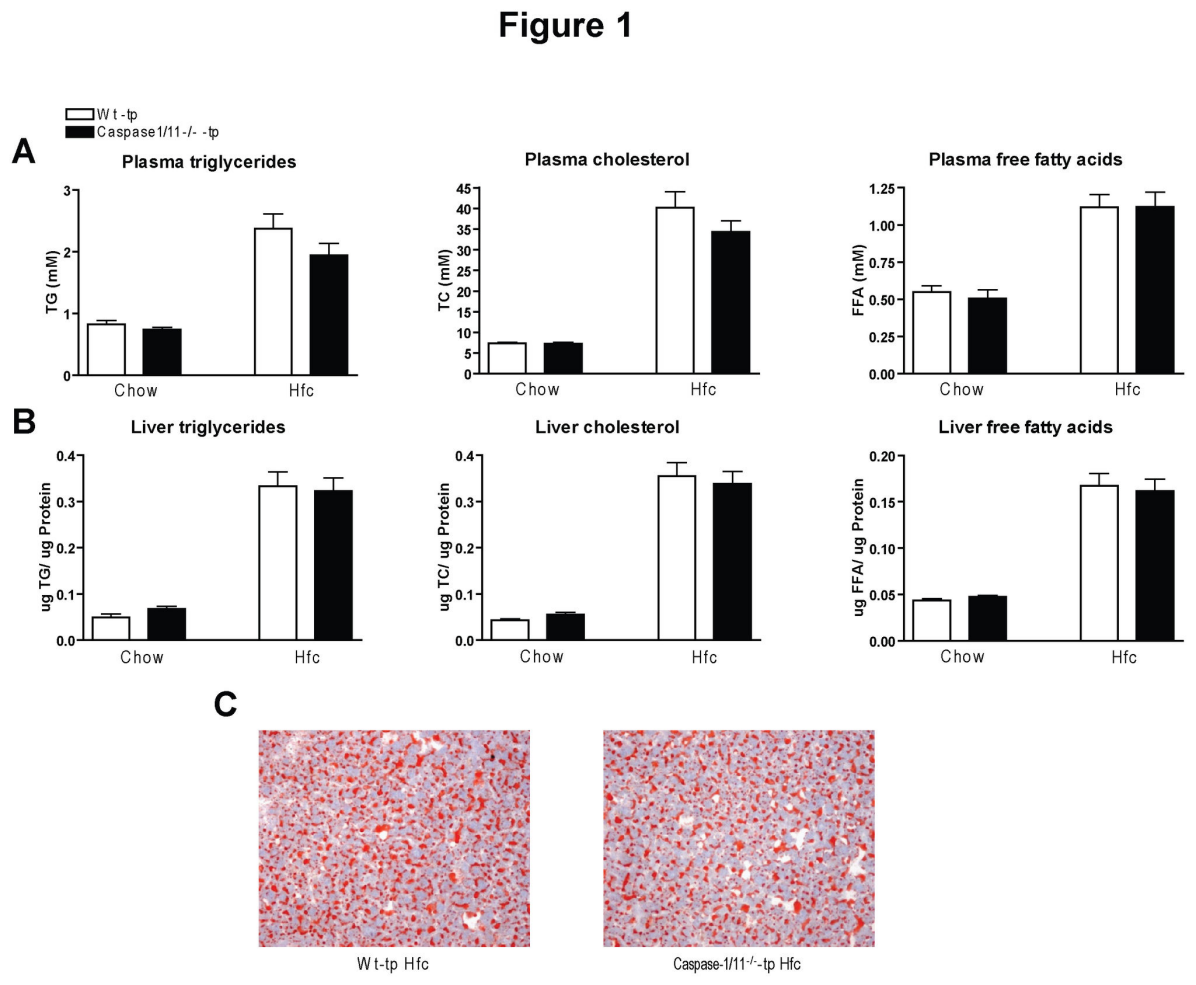
Plasma and liver lipid levels. (*A*) Plasma total triglycerides (TG), total cholesterol (TC) and free fatty acids (FFA) after 3 months on a chow or HFC diet in Wt-tp and caspase-1/11^-/-^-tp mice. (*B*) Liver TG, TC and FFA after 3 months on chow or HFC diet. (C) Representative images (100x magnification) of the Oil Red O staining of liver sections from Wt-tp and caspase-1/11^-/-^-tp mice on HFC for 3 months. Data are represented as mean +/- SEM.

As expected, the levels of triglycerides (TG), total cholesterol (TC) and free fatty acids (FFA) in both plasma and liver were higher after 3 months of HFC feeding than after 3 months of chow diet. However, no differences in plasma lipid concentrations were found between Wt-tp and caspase-1/11^-/-^-tp mice on HFC diet (Figure 1A). In line with these data, HFC feeding resulted in equal levels of steatosis in the two transplanted groups, as indicated by biochemical measurements of lipids in the liver (Figure 1B) and oil red O staining (Figure 1C). No difference in weight between the different groups was observed. Taken together, these data demonstrate that hematopoietic inflammasome activation has no effect on lipid concentrations in plasma and liver.

### Less hepatic inflammation in caspase-1/11^-/-^-tp mice on HFC diet

**Figure 2:**
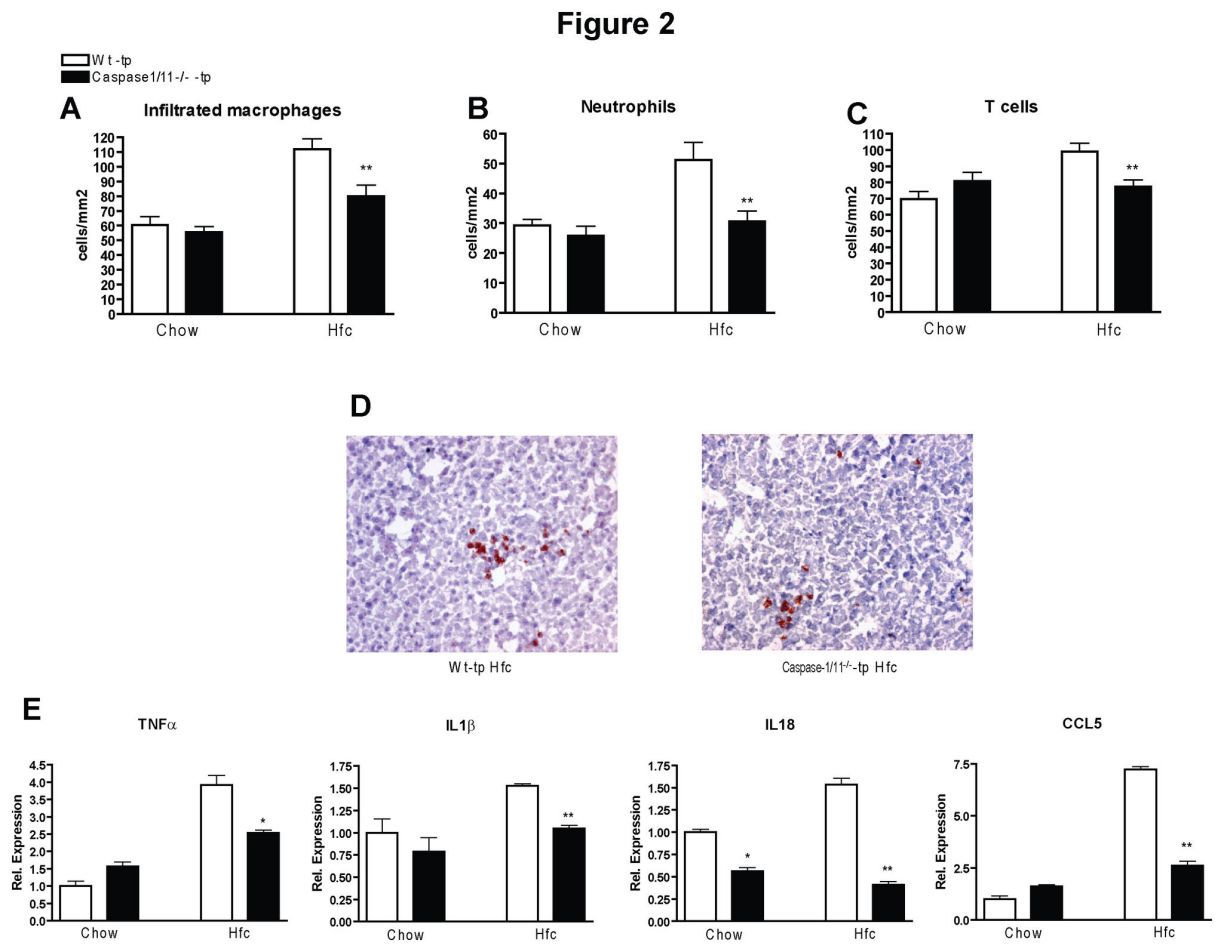
Parameters of hepatic inflammation. (*A*-*C*) Liver sections were stained for infiltrated macrophages and neutrophils (Mac-1), neutrophils (NIMP) and T cells (CD3^+^). (*D*) Representative pictures of Mac-1 staining (200x magnification) after 3 months of HFC feeding in Wt-tp and caspase-1/11^-/-^-tp mice. (*E*) Gene expression of pro-inflammatory cytokines tumor necrosis factor alpha (TNFα), interleukin 1 beta (IL1β), interleukin 18 (IL18) and chemokine (C-C motif) ligand 5 (CCL5) in isolated KCs. Data are represented as mean +/- SEM and were set relative to the Wt-tp group on chow diet. * and ** Significantly different from Wt-tp on HFC diet. *p<0.05, **p<0.01.

To determine the effect of caspase-1 and caspase-11 deletion in KCs on hepatic inflammation during the development of NASH, we performed immunohistochemical analysis using inflammatory markers. Scoring of stained sections for infiltrated macrophages (Mac1) (p=0.007), neutrophils (NIMP) (p=0.0043) and T cells (KT3) (p=0.0028) revealed there to be less hepatic inflammation in caspase-1/11^-/-^-tp mice than in Wt-tp mice on HFC diet (Figure 2A, B, C). Clustering of inflammatory cells, when present, was also more pronounced in the livers of Wt-tp mice than in those of caspase-1/11^-/-^-tp animals (Figure 2D). 

To determine the direct contribution of inflammasome activation in KCs to the inflammatory response in the liver, we analyzed inflammatory gene expression in isolated KCs from both transplanted groups. Isolated KCs from caspase-1/11^-/-^-tp mice had lower expression of *Tnf*-*a* (p=0.0421), *IL-1β* (p=0.007), *IL18* (p=0.005) and *Ccl5* (p=0.003) than those from Wt-tp mice on HFC diet (Figure 2E). These gene expression data support our histological findings and confirm that the milder hepatic inflammation observed in caspase-1/11^-/-^-tp mice is a result of caspase-1 and caspase-11 deletion, specifically in KCs.

Although fibrosis development was only moderate, scoring of the Sirius Red-stained liver sections revealed collagen content to be lower in the livers of caspase-1/11^-/-^-tp mice than in those of Wt-tp mice (p=0.0481) (Figure S1A-C). However, hepatic gene expression of the fibrosis-related genes transforming growth factor β (*Tgf-β*) and collagen 1A1 (*Col1a1*) was not significantly different between the transplanted groups (Figure S1D). No differences in plasma ALT levels were observed between Wt-tp and caspase-1/11^-/-^-tp mice (Figure S1E).

Despite no difference in the foamy appearance of Kupffer cells, caspase-1/11^-/-^-tp mice have reduced lysosomal dysfunction and less cholesterol crystallization than Wt-tp mice on HFC. 

**Figure 3:**
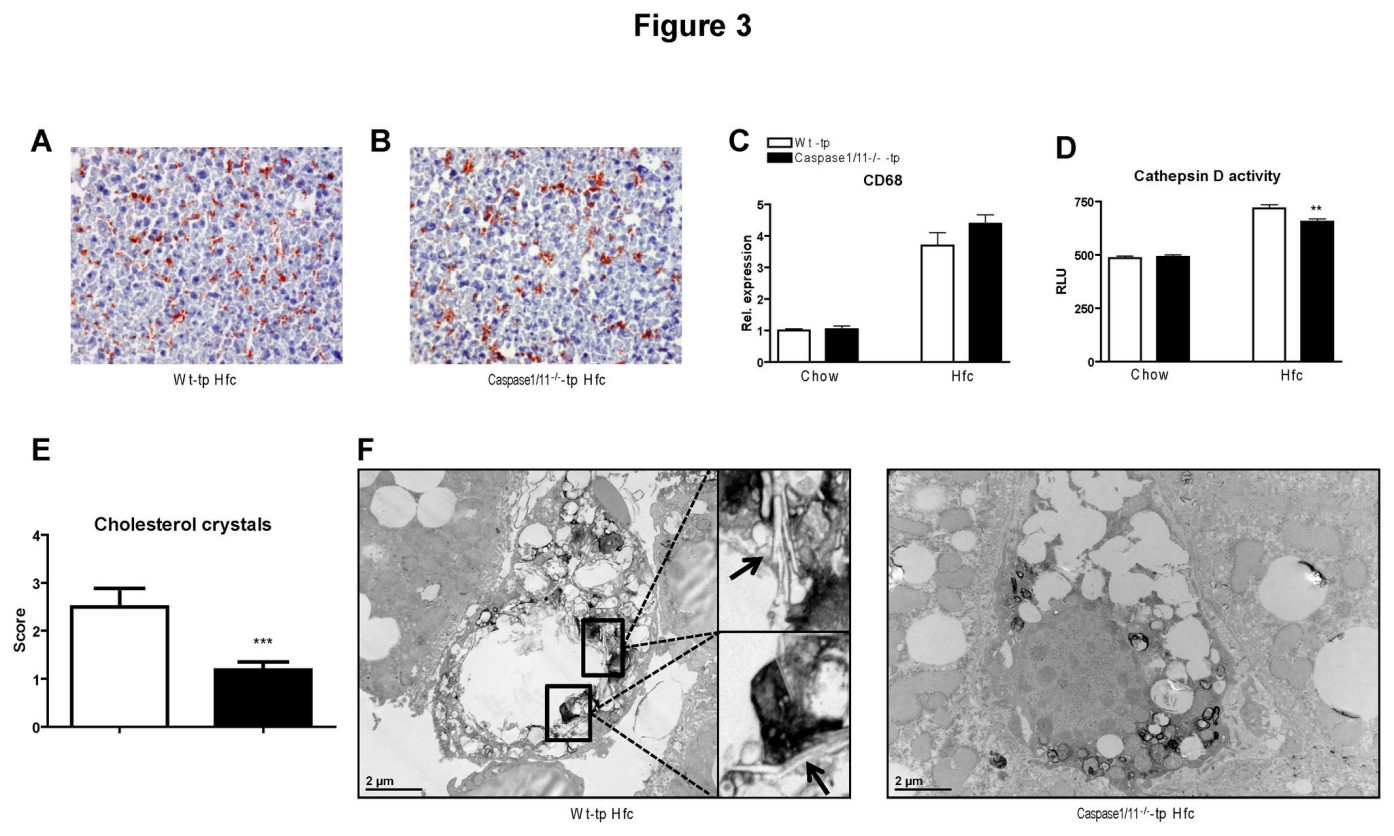
Foamy appearance of Kupffer cells, Cathepsin D activity and electron microscopy of KCs. (*A*, *B*) Representative pictures of CD68 staining (200x magnification) after 3 months of HFC feeding in Wt-tp and caspase-1/11^-/-^-tp mice, respectively. (*C*) Gene expression analysis of macrophage and Kupffer cell marker Cd68 in liver of Wt-tp and caspase-1/11^-/-^-tp mice. (*D*) Activity of the lysosomal enzyme cathepsin D in livers of Wt-tp and caspase-1/11^-/-^-tp mice. (*E*) Scoring for the amount of cholesterol crystallization inside Kupffer cells; 0 indicates that no cholesterol crystals were present while 5 indicates the highest amount of cholesterol crystals present. (*F*) Representative electron microscopy pictures of KCs from Wt-tp and caspase-1/11^-/-^-tp mice on HFC diet. Lysosomes are indicated in black by acid phosphatase staining and cholesterol crystals are indicated by arrows. Data are represented as mean +/- SEM and were set relative to the Wt-tp group on chow diet. *Significantly different from Wt-tp on HFC diet. *p<0.05.

To determine whether inflammasome activation affects the foamy appearance of KCs, staining of liver sections against CD68, a macrophage marker that stains KCs, was performed. The comparison of CD68 positive cells revealed no difference between Wt-tp and caspase-1/11^-/-^-tp animals (Figure 3A+B). These data were confirmed by gene expression analysis of *Cd68*, which also showed an increase after 3 months of HFC diet and no difference between both transplanted groups upon HFC (Figure 3C). Additionally, no difference in steatosis was observed (data not shown). These data suggest that inflammasome activation has no effect on the foamy appearance of KCs.

To assess lysosomal dysfunction, the hepatic activity of the lysosomal enzyme cathepsin D was measured. In line with the differences in hepatic inflammation, the livers of caspase-1/11^-/-^-tp mice had less cathepsin D activity than those of Wt-tp mice on HFC diet (Figure 3D) (p=0.0042). Despite a significant effect, the difference in cathepsin D activity is small and it is not clear whether it is biological relevant. Since lysosomal dysfunction and disturbed lysosomal enzyme activity is associated with the presence of cholesterol crystals, electron microscopy was performed to study any differences in KC structure and cholesterol crystallization. As demonstrated in Figure 3E+F, KCs of caspase-1/11^-/-^-tp mice on HFC diet had considerably fewer cholesterol crystals than KCs of Wt-tp mice. Scoring electron microscopy pictures from 30 Kupffer cells from each experimental group revealed that caspase-1/11^-/-^-tp mice had significantly less cholesterol crystals inside their Kupffer cells than Wt-tp mice after 3 months of HFC (Figure 3E). These data indicate that inflammasome activation stimulates cholesterol crystallization and is associated with disturbed lysosomal function.

### Caspase-1/11^-/-^-tp mice have increased cholesterol efflux and restored autophagy compared to Wt-tp mice on HFC

**Figure 4:**
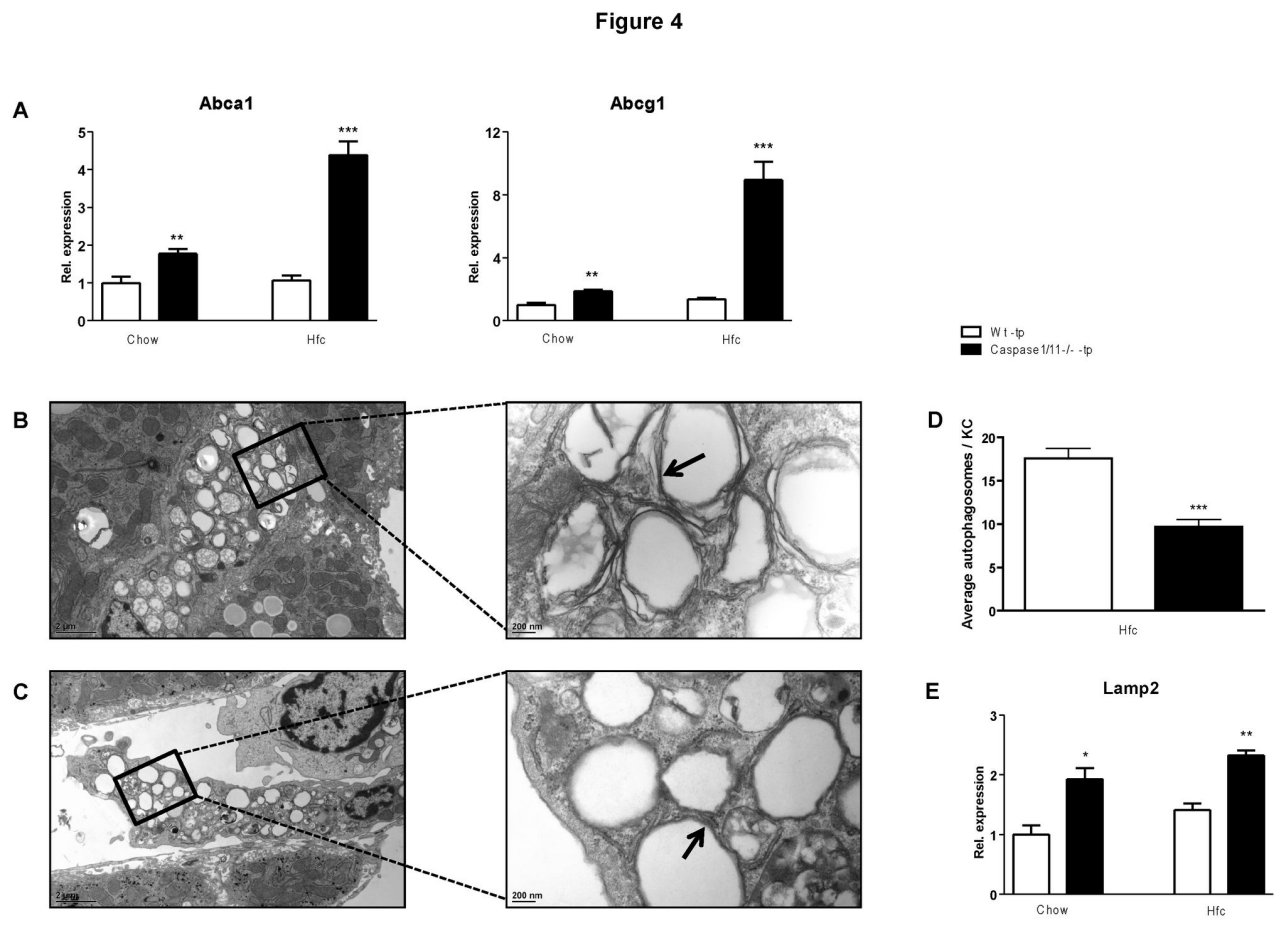
Parameters for cholesterol efflux and autophagy. (*A*) Gene expression analysis of ABC-transporter a1 (Abca1) and ABC-transporter g1 (Abcg1) in isolated KCs. (*B*, *C*) Representative electron microscopy images of KCs from Wt-tp (*B*) and caspase-1/11^-/-^-tp (*C*) mice on HFC diet. Double membranes surrounding lipid droplets (autophagosomes) are indicated by arrows. (*D*) Average autophagosome number per Kupffer cell. (*E*) Gene expression analysis of lysosomal-associated membrane protein 2 (Lamp2) in isolated KCs. Data are represented as mean +/- SEM and were set relative to the Wt-tp group on chow diet. *Significantly different from Wt-tp on HFC diet. **p<0.01, ***p<0.001.

ATP binding cassette transporter a1 (Abca1) and ATP binding cassette transporter g1 (Abcg1) are two well-known cholesterol transporters responsible for cholesterol efflux. To investigate whether the stimulation of cholesterol crystallization by caspase-1 is related to cholesterol efflux, gene expression analysis of *Abca1* and *Abcg1* in isolated KCs was performed. On both chow and HFC diet, expression of *Abca1* (p=0.0099; p=0.0001) and *Abcg1* (p=0.0025; p=0.0006) was increased in KCs of caspase-1/11^-/-^-tp mice compared to Wt-tp mice (Figure 4A). Interestingly, in KCs of caspase-1/11^-/-^-tp mice on HFC diet, increased expression of *Abca1* (p=0.0005) and *Abcg1* (p=0.0009) was found compared to mice on chow diet, whereas HFC diet had no effect in KCs of Wt-tp mice (Figure 4A). 

To investigate whether the decreased cholesterol efflux in KCs of caspase-1/11^-/-^ -tp mice is related to disturbed autophagy, electron microscopy was used to quantify the number of lipid-containing autophagosomes present inside KCs (Figure 4B+C). We determined the average amount of autophagosomes per KC from each transplanted group and found that the higher degree of cholesterol crystallization observed in Wt-tp mice on HFC diet is accompanied by a higher average autophagosome number per KC relative to caspase-1/11^-/-^-tp mice on HFC diet (Figure 4D) (p<0.001), indicative of abnormal autophagy. Furthermore, expression of *Lamp2* in isolated KCs from caspase-1/11^-/-^-tp mice was increased compared to Wt-tp mice, both on chow (p=0.0181) and HFC diet (p=0.0094) (Figure 4E). Altogether, these data indicate that increased hepatic inflammation and cholesterol crystallization in caspase-1/11^-/-^-tp mice is associated with disturbed cholesterol efflux and affected autophagic activity in KCs.

**Figure 5:**
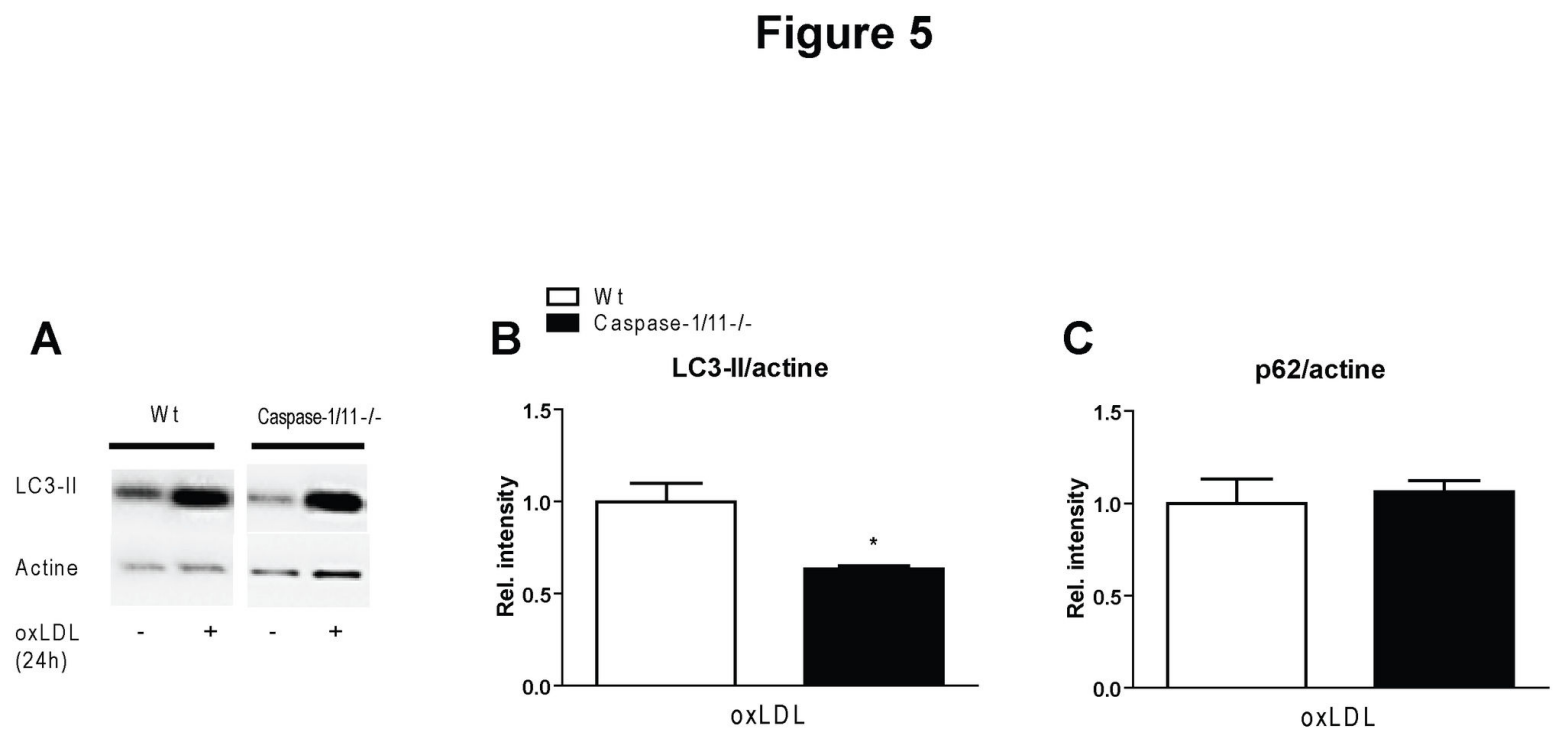
Autophagy in Wt and caspase-1/11^-/-^ bone marrow derived macrophages. (*A*) Western blots of LC3-II in Wt and caspase-1/11^-/-^ bone marrow derived macrophages (BMDM) with and without oxLDL incubation for 24h. (*B*, *C*) Quantification of western blot for LC3-II and p62 levels in BMDM from Wt and caspase-1/11^-/-^ mice after oxLDL incubation (24h), corrected for actine levels. Data from three separate experiments are represented as mean +/- SEM and were set relative to Wt BMDM. *Significantly different from Wt BMDM. *p<0.05.

To further examine the effect of inflammasome activation on autophagy in macrophages, bone marrow derived macrophages (BMDM) from Wt and caspase-1/11^-/-^ mice were incubated with oxLDL for 24h. Incubation with oxLDL increased LC3-II levels in macrophages from Wt and caspase-1/11^-/-^ mice (Figure 5A). After 24h oxLDL incubation in Wt macrophages, levels of LC3-II increased to a higher extent than in caspase-1/11^-/-^ macrophages (Figure 5B), while no difference was observed in levels of p62 (Figure 5C), suggesting that although some components of the autophagic process are not affected, the final fusion/degradation step with the lysosome is impaired. These data further strengthen our *in vivo* findings that inflammasome activation in macrophages is associated with affected autophagic activity.

## Discussion

In the present study, we investigated whether inflammasome activation in Kupffer cells stimulates the formation of cholesterol crystals and thereby contributes to the inflammatory response during NASH. Our results indeed indicate for the first time an important role for inflammasome activation in Kupffer cells in stimulating cholesterol crystallization, thereby triggering hepatic inflammation. Our data suggest that insufficient cholesterol efflux and disturbed autophagy are two important mechanisms underlying the formation of inflammasome mediated cholesterol crystals. Inflammasomes thus contribute to a vicious cycle whereby, upon activation, new crystals are formed which then further enhance inflammasome activation. 

### Inflammasome activation in KCs plays a major role in hepatic inflammation

Despite much effort, the exact role of inflammasome activation during the development of NASH remains poorly understood. Recent studies describing the role of caspase-1 and caspase 11 during NASH produced conflicting results. On the one hand, when compared with control mice, caspase-1/11^-/-^ mice receiving a methionine and choline-deficient (MCD) diet for 6 weeks were observed to have lower expression levels of genes involved in inflammation and fibrosis [14]. In line with our findings, *Dixon et al*. also showed that KC depletion by clodronate injection suppressed caspase-1 activation and reduced fibrogenesis, suggesting that KCs are the most important cellular source of active caspase-1 during MCD-induced NASH [14]. In another recent study from the same group, it was shown that mice deficient for caspase-1 and caspase-11 were protected from high fat-induced steatosis, inflammation and early fibrogenesis [15]. Conversely, another study showed that the inflammasome negatively regulates NASH progression. Here, caspase-1/11^-/-^ mice fed an MCD diet for 4 weeks had higher levels of ALT and AST, as well as more microvesicular and macrovesicular hepatic steatosis, and more hepatic inflammation than their C57Bl6 wild-type controls fed the same diet [16]. Additional evidence came from an experiment in the same study in which bone marrow from *Nlrp3* ^*-/-*^ or *Asc* ^*-/-*^ mice was transplanted into wild-type mice, from which the authors concluded that ablation of these genes in hematopoietic cells has no effect on hepatic inflammation after MCD feeding [16].

Although the reason for these contradictive findings is not clear, a difference in gut microbiota or in housing conditions are fitting explanations. For example, gut microbiota are known to undergo changes in obese individuals, as well as in obese mouse models [17], and diet is known to influence gut microbiota composition in both animal models and humans [18,19]. It is therefore reasonable to hypothesize that the contradiction regarding the role of inflammasome activation in hematopoietic cells may be related to the fact that we used an HFC diet while *Henao-Mejia et al*. used an MCD diet. After all, different dietary regimes can lead to diverse inflammatory effects: MCD feeding results in more acute inflammation and severe liver damage, whereas mice fed an HFC diet have more chronic low-grade hepatic inflammation. It is therefore possible that the causes of inflammasome activation depend on the specific type and trigger of inflammation.

Another explanation for the observed differences could be related to the fact that the present study used hyperlipidemic *Ldlr* ^*-/-*^ mice with a human-like lipid profile while the other study used normolipidemic wild-type (C57Bl6) mice. The drastic differences between these mice with regard to plasma lipid levels may also affect activation of the inflammasome via the effect on autophagy for example. Increased lipid levels have been shown to repress autophagic function [20], thereby possibly enhancing IL-1b secretion as autophagy is able to control IL-1b production [21–23]. 

While we have investigated the inflammasome in relation to NASH, others have studied its relation to atherosclerosis. Our current data are in line with most findings regarding the role of inflammasomes in atherosclerosis [24,25]. Studies have shown that *ApoE* ^*-/-*^ *Caspase-1/11* ^*-/-*^ double deficient mice have fewer signs of atherosclerosis than *ApoE* ^*-/-*^ mice, meaning that inflammasome activation is also a key factor in the inflammatory response during atherosclerosis [24,25]. It is also important to note that, in the same model as used here, *Ldlr* ^*-/-*^ mice reconstituted with bone marrow from *Nlrp3* ^*-/-*^ or *Asc* ^*-/-*^ mice had less atherosclerosis after an HFC diet, indicating a reduced inflammatory response during disturbed inflammasome activation and thereby confirming our results [5]. We suggest that the involvement of inflammasomes in inflammation is influenced by the status of immune responses, as well as by the specific disease models investigated. Our data support the strong involvement of inflammasome activation in hematopoietic cells, especially KCs, in the context of hepatic inflammation. Of note, it is important to keep in mind that in addition to Kupffer cells, other hematopoietic cells are also lacking caspase-1 and caspase-11 in the caspase-1/11^-/-^-tp mice. These cells secrete myeloid precursors and as such can directly contribute to the reduction in the inflammatory response [26]. Moreover, KCs can also indirectly influence the phenotype of neighboring hepatocytes and other immune cells via the production of inflammatory cytokines such as TNF and interleukins, and cross-talking with other liver cell types [27]. Therefore, it is very likely that in addition to Kupffer cells, other liver cells modulate directly and indirectly the hepatic intracellular inflammatory signaling pathway.

### Inflammasome activation promotes cholesterol crystallization via reduced cholesterol efflux

During atherosclerosis, intracellular lipid accumulation in foam cells can result in the formation of cholesterol crystals [28]. Since atherosclerosis and NASH share similar hallmarks [29], it is likely that cholesterol is also crystallized inside KCs during NASH development. Indeed, previously we confirmed that cholesterol crystallization in KCs is associated with hepatic inflammation in hyperlipidemic *Ldlr* ^*-/-*^ mice fed an HFC diet [30]. Additionally, it was recently shown that also human NASH patients have cholesterol crystallization and the formation of “crown-like” structures in activated KCs. In this way they were able to distinguish NASH from simple steatosis, as in these patients cholesterol crystals were not formed [31]. Others have shown that crystalline structures activate the inflammasome by causing lysosomal damage [32,33] and that cholesterol crystals can lead to lysosomal rupture and subsequently the release of cathepsin B and the generation of ROS, both described as activators of the inflammasome [34]. In the current study, we have demonstrated for the first time that inflammasome activation contributes to the promotion of cholesterol crystallization in the liver.

Although the exact mechanisms behind this are not yet clear, we propose that reduced cholesterol efflux and disturbed autophagy, the process by which cytoplasmic components are broken down and recycled, represent an important link between lysosomal cholesterol accumulation, inflammasome activation and cholesterol crystal formation. From literature it is known that reduced cholesterol efflux is associated with foam cell formation, whereas increased cholesterol crystals are found in foamy macrophages. In macrophages, cholesterol efflux via ABCA1 and ABCG1 prevent the excessive accumulation of lipids, thereby protecting against the formation of atherosclerotic lesions [35]. Moreover, animal studies show that in transgenic mice disruption of ABCA1 gene induces atherosclerosis [36], in which it was shown that cholesterol crystals activate the inflammasome [5]. 

### Disturbed autophagy as driver for hepatic inflammation

We propose that autophagy is dysfunctional as indicated by increased levels of both LC3-II and p62 in our Wt macrophages in our in vitro set-up. Due to the fact that LC3-II and p62 are both degraded in the autolysosome, the lysosomal-dependent turnover of these proteins has emerged as a measure of autophagic flux [37]. Thus, accumulation of LC3-II and p62 indicates autophagic flux inhibition at any point beyond autophagosome formation, while increased p62 alone indicates reduced autophagy [37]. Therefore, our novel findings indicate that autophagy activity in KCs is affected upon HFC and may therefore lead to hepatic inflammation in this way. Additionally, it was recently highlighted that cholesterol efflux by lipid-loaden macrophages is dependent on autophagy, as macrophages with impaired autophagy were not able to clear accumulated cholesterol *in vivo*. Furthermore, autophagy-mediated cholesterol efflux was shown to be primarily Abca1 dependent [38]. These data can explain our observations regarding reduced cholesterol efflux and abnormal autophagy, as shown by the accumulation of autophagosomes and reduced *Lamp2* expression in KCs. Lamp2 deficiency was previously shown to be associated with accumulation of autophagic vacuoles in different tissues such as muscle, heart and liver [39]. In humans, a mutated Lamp2 gene results in Danon disease, characterized by intracytoplasmic vacuoles containing autophagic material and glycogen in skeletal and cardiac muscle cells [40]. Since autophagy is disturbed in lysosomal storage disorders [41] and dysfunctional autophagy results in inflammasome activation and increased accumulation of cholesterol crystals [42], further increases in cholesterol crystallization may result in a vicious cycle of inflammation through further activation of the inflammasome. Such a mechanism might explain the sustained inflammatory response seen in the liver.

A number of other studies provide further evidence that disturbed autophagy may also contribute to hepatic inflammation. Firstly, autophagy has been shown to control IL-1b production by targeting pro-IL-1b for lysosomal degradation and by regulating NLRP3 inflammasome activation [23]. Secondly, it has been shown to regulate hepatocyte lipid metabolism, a process called lipophagy [20,43]. Taken together with our current findings this suggests a link between abnormal autophagy, inflammasome activation and cholesterol crystallization during the development of NASH.

In summary, our data demonstrate that inflammasome mediated cholesterol crystallization in KCs is an important factor during the progression of hepatic inflammation in NASH. Of note, in addition to caspase-1, the mice that we have used are also lacking caspase-11. Caspase-11 was found to be important in non-canonical inflammasome activation, which is mainly involved in inflammation driven by gram-negative bacteria [44]. As inflammasome activation by cholesterol crystals is known to be caspase-1 dependent [45], it is most likely that caspase-11 plays a minor role in this process. Nevertheless, as it was shown that caspase-11 is involved in inflammation and autophagosome autophagosome fusion [44,46], we cannot exclude the possibility that caspase-11 is also playing a role in cholesterol crystallization and triggering hepatic inflammation during NASH. More in general, blocking inflammasome activation in KCs can be seen as a potential target for therapy options in obesity related diseases. Future studies should focus on the mechanisms that link autophagy, inflammasomes and cholesterol crystals in relation to the development of hepatic inflammation. 

## Supporting Information

Figure S1 **Parameters of fibrosis.** (*A*, *B*) Representative images (200x magnification) of Sirius Red positive sections after 3 months of HFC diet in Wt-tp and caspase-1/11^-/-^-tp mice, respectively. (*C*) Quantification of the Sirius Red staining. (*D*) Gene expression analysis of transforming growth factor beta (Tgf-β) and collagen 1A1 (Col1a1) in whole liver. (*E*) ALT measurements in plasma from Wt-tp and caspase-1/11^-/-^-tp mice. Data were set relative to the Wt-tp group on chow diet. *Significantly different from Wt-tp on HFC diet. *p<0.05.
(TIF)
