# Supplementary figures and images for: Macrophage Specific Caspase-1/11 Deficiency Protects against Cholesterol Crystallization and Hepatic Inflammation in Hyperlipidemic Mice
Source: PLoS One. 2013 Dec 2;8(12):e78792. doi: 10.1371/journal.pone.0078792 (PMC3846469; doi:10.1371/journal.pone.0078792)

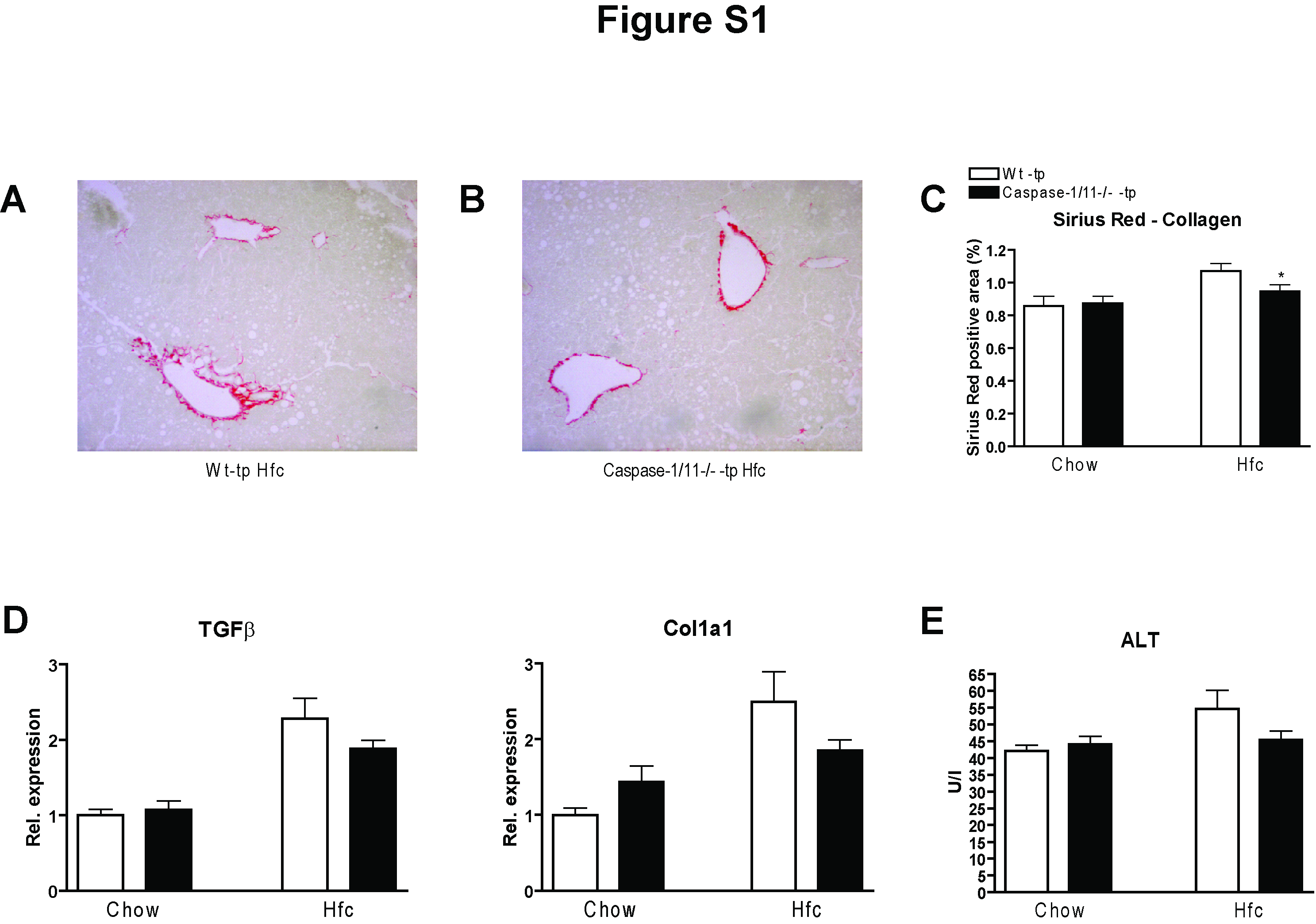

Supplement: Figure S1 — Parameters of fibrosis. (A, B) Representative images (200x magnification) of Sirius Red positive sections after 3 months of HFC diet in Wt-tp and caspase-1/11-/--tp mice, respectively. (C) Quantification of the Sirius Red staining. (D) Gene expression analysis of transforming growth factor beta (Tgf-β) and collagen 1A1 (Col1a1) in whole liver. (E) ALT measurements in plasma from Wt-tp and caspase-1/11-/--tp mice. Data were set relative to the Wt-tp group on chow diet. *Significantly different from Wt-tp on HFC diet. *p<0.05. (TIF) [file pone.0078792.s001.tif]
